# The Ratio of CD226 and TIGIT Expression in Tfh and PD-1^+^ICOS^+^Tfh Cells Are Potential Biomarkers for Chronic Antibody-Mediated Rejection in Kidney Transplantation

**DOI:** 10.1155/2022/5326083

**Published:** 2022-06-12

**Authors:** Ji-wen Fan, Yu Fan, Zheng-li Wan, Lin Yan, Ya-mei Li, Yang-juan Bai, Lan-lan Wang, Jie Chen, Yi Li

**Affiliations:** ^1^Department of Laboratory Medicine, West China Hospital, Sichuan University, Chengdu 610041, China; ^2^Department of Urology, National Clinical Research Center for Geriatrics and Organ Transplantation Center, West China Hospital of Sichuan University, No. 37 Guoxue Xiang, Chengdu 610041, China; ^3^Department of Urology, West China School of Nursing, Sichuan University, No. 37 Guoxue Xiang, Chengdu 610041, China

## Abstract

Kidney transplantation is the ideal treatment for end-stage renal disease (ESRD). Chronic antibody-mediated rejection (CAMR) is the main cause of graft failure. Tfh and B cells are key immune cells that play important roles in CAMR. In this study, the populations of different Tfh cell phenotypes and B cell subsets in CAMR were investigated in a total of 36 patients. Based on Banff-2019, 15 patients were diagnosed with CAMR (CAMR group), 11 recipients were diagnosed with recurrent or de novo IgA nephropathy (IgAN group), and 10 patients displayed stable renal function (stable group). The Tfh and B cell subsets were analyzed by flow cytometry. The percentage and absolute number of PD-1^+^ICOS^+^Tfh cells were significantly higher in CAMR (*p* < 0.05), as was the ratio of CD226^+^Tfh cells to TIGIT^+^Tfh cells (*p* < 0.05). Compared with stable recipients, CAMR patients had lower naïve B cells and higher unswitched memory B cells, which were also significantly related to renal function (*p* < 0.05). Using the logistic regression model, we concluded that the estimated glomerular filtration rate (eGFR), absolute number of PD-1^+^ICOS^+^Tfh cells, and ratio of CD226^+^Tfh cells to TIGIT^+^Tfh cells were independent risk factors for CAMR. The combination of eGFR, PD-1^+^ICOS^+^Tfh cells, and the ratio of CD226^+^Tfh cells to TIGIT^+^Tfh cells showed better diagnostic efficacy for CAMR than each single parameter. The collective findings show that monitoring different Tfh phenotypes and B cell subsets is beneficial to kidney transplant recipients and implicate the combination of eGFR, number of PD-1^+^ICOS^+^Tfh cells, and ratio of CD226^+^Tfh cells to TIGIT^+^Tfh cells as a biomarker for diagnosing CAMR. The findings may also inform new strategies to identify and treat CAMR.

## 1. Introduction

Kidney transplantation is one of the best treatments for chronic kidney disease and end-stage renal disease (ESRD). According to the OPTN/SRTR 2019 Annual Data Report, the number of kidney transplants has increased each year since 2015, reaching the highest annual count of 24,273 in 2019 [1]. With the development of technologies and the application of novel immunosuppressants, the short-term living-donor allograft survival rate has improved significantly. However, kidney allograft failure remains a serious condition as there are many causes of graft failure, which are heterogeneous, multifactorial, and time-dependent [2]. Among these, chronic antibody-mediated rejection (CAMR) is the major cause of graft failure after kidney transplantation (KT) [3]. Some problems need to be addressed. First, the gold standard for CAMR is biopsy, which is an invasive procedure that depends on tissue quality. Second, effective treatment for CAMR remains an unsolved problem. Therefore, it is important to identify a potential target for the diagnosis and treatment of long-term graft survival.

CAMR involves continuous exposure to donor antigens and the production of donor-specific antibodies (DSA) that attack the graft. During CAMR, naïve B cells exposed to antigens differentiate into DSA-specific plasma cells through germinal centers (GCs). In the GCs, antigen-specific B cells produce affinity mature antibodies with specific effector functions through somatic high mutation and class switch recombination of their immunoglobulin (Ig) genes [4]. This process involves the initial uptake of donor antigens and the response of surface presenting cells (APCs) to donor antigens. This leads to activation of follicular helper T cells (Tfhs) and continuous promotion of class-switching naïve B cells and memory B cells into plasma cells [5]. Thus, long-lived plasma B cells and DSA are persistent and eventually lead to the development of CAMR. Monitoring B cells in kidney transplant patients might be important and beneficial [6, 7].

As a trigger for B cell antibody production, Tfh cells play a key role in promoting the appearance of DSA [8]. Tfh cells are antigen-experienced CD4^+^ T cells that provide critical help to B cells within GCs of secondary lymphoid organs. Tfh cells are characterized by the expression of C-X-C chemokine receptor 5 (CXCR5), which is necessary for the response to C-X-C chemokine ligand 13 (CXCL13) in B cell follicles. Tfh cells can drive B cell activation, proliferation, and selection of high-affinity clones and differentiate into long-life plasma cells or memory B cells by chemokines or cytokines, such as interleukin-21 (IL-21) [8, 9]. Different Tfh statuses have been described with some inhibitory cell surface molecules, such as programmed cell death protein 1 (PD-1) and T cell immunoreceptor with Ig and ITIM domains (TIGIT), and some active cell surface molecules, such as inducible costimulatory molecule (ICOS) and CD226. [10–14]. By establishing a mouse kidney transplantation model, researchers observed donor-reactive CD4^+^ CXCR5^+^ circulating Tfh cells, which provide B cells to help produce antibodies expand after allotransplantation and which display an activated ICOS^+^PD-1^+^ phenotype after transplantation [15]. Louis et al. found that circulating Tfh cells and activated B cells were increased in KTRs with CAMR. These circulating Tfh cells produced large amounts of IL-21 in response to donor antigen stimulation and induced B cells to differentiate into antibody-secreting cells that produce DSA [16]. In our previous study, we found that an elevated ratio of circulating Tfh to T follicular regulatory (Tfr) cells was associated with chronic allograft dysfunction (CAD) [17].

TIGIT is an immune inhibitory receptor present on some T cells and natural killer (NK) cells. CD226, which is expressed on the surface of NK cells, monocytes, and subsets of T cells, is involved in immune activation. TIGIT and CD226 compete for binding to the same ligand, CD155, on APCs [18–20]. The expression levels of TIGIT and CD226 depend on T cell subsets and their activation status. TIGIT was reportedly highly expressed in Tfh cells, and CD226 signals promoted cell differentiation in the early stages of Tfh in humans [14, 21]. One study showed that TIGIT^+^Tfh cells exhibited strong B cell help function, while another study showed that TIGIT expression was associated with a decrease in Tfh cell proliferation [14, 22]. Recently, an increasing number of studies have demonstrated the importance of the TIGIT/CD226 axis in tumors and autoimmune diseases [23, 24]. Soluble CD226 molecules can be used as serum markers for cancer diagnosis and prognosis [25]. Akiyama et al. found that peripheral TIGIT^+^Tfh cell populations produced high levels of IL-21 in IgG4-related diseases, reflecting disease activity [26]. In COVID-19 patients, the number of CD226^+^Tfh cells was significantly reduced within 1 week of recovery [27]. However, the roles of TIGIT and CD226 in Tfh cells in CAMR remain unknown.

This study is aimed at investigating the different Tfh cell phenotypes and B cell subset distributions in the peripheral blood in CAMR. The potential of these cells as biomarkers for diagnosis was analyzed.

## 2. Materials and Methods

### 2.1. Patient Cohort

A total of 36 patients who underwent treatment or long-term follow-up at the West China Hospital of Sichuan University from October 2020 to July 2021 were included in this study. Basic patient information, including age, sex, body mass index (BMI), transplant time, biochemical test results, and pathological biopsy results were collected. Based on Banff-2019, 15 patients were diagnosed with CAMR (CAMR group), 11 patients were diagnosed with recurrent or de novo IgA nephropathy (disease control group, IgAN group), and 10 patients had stable renal function (stable group). All study procedures were approved by the Research Ethics Committee of West China Hospital of Sichuan University, China. Written informed consent was obtained from all participants before their inclusion in the study.

### 2.2. Flow Cell Cytometry

**Figure 1:**
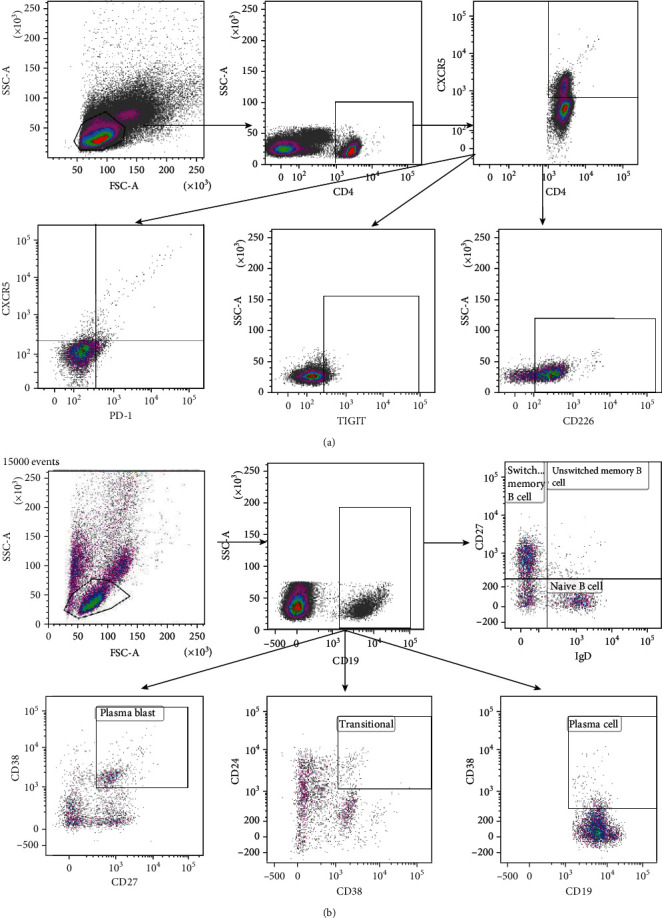
Gating strategy for analysis using logic gates. Measurements were performed with fresh blood samples. (a) Tfh cells were defined as CD4^+^CXCR5^+^ T cells in the lymphocyte population. The Tfh cell phenotypes were PD-1^+^ICOS^+^CD4^+^CXCR5^+^ T cell (PD-1^+^ICOS^+^Tfh cells), CD226^+^CD4^+^CXCR5^+^ T cell (CD226^+^Tfh cells), and TIGIT^+^CD4^+^CXCR5^+^ T cell (TIGIT^+^Tfh cells). (b) B cells were further defined as CD19-expressing cells in the lymphocyte population. CD19^+^ B cells were analyzed for the expressions of IgD and CD27, CD24, and CD38. The B cell populations included CD27^−^IgD^+^ (naïve B cells), CD27^+^IgD^+^ (unswitched memory B cells), CD27^+^IgD^−^ (switched memory B cells), CD24^++^CD38^++^ (transitional B cells), CD27^+^CD38^+^ (plasmablasts), and CD38^+^ B cells (plasma cells).

Fresh peripheral blood samples were collected in 6 ml heparin tubes (BD Biosciences, San Jose, CA, USA) and processed within 1 h after drawing blood. Peripheral blood mononuclear cells (PBMCs) were isolated using Ficoll (Solarbio Life Science, Beijing, China). Surface staining of PBMCs was performed using the following monoclonal antibodies (mAbs): CD19-phycoerythrin (PE), CD27-PE-cy7, CD38-APC, IgD-fluorescein isothiocyanate (FITC), CD24-APC-eF780, CD4-APC-eF780, CXCR5-APC, PD-1-BV510, ICOS-BV421, TIGIT-FITC, and CD226-PE-Cy7. All flow cytometry analyses were performed on a FACS Canto II instrument (BD Biosciences). Results were analyzed using the Kaluza V2.1 software. The gating strategies are illustrated in Figure 1.

### 2.3. Laboratory Analyses

Serum creatinine (Scr) levels were determined using the picric acid method (Roche Diagnostics, Mannheim, Germany). The Modification of Diet in Renal Disease formula adjusted for the Chinese population was used to calculate the estimated glomerular filtration rate (eGFR) [28].

eGFR (ml/min/1.73 m^2^) = 186 × Scr (mg/dl) − 1.154age − 0.203 × (0.742 if female) × 1.233 cystatin C (Cys − c) was determined using a turbidimetric inhibition immuno assay (Roche Diagnostics).

### 2.4. Detection of Anti-HLA Antibody

Serum samples from recipients before and after transplantation were collected to detect anti-HLA class I (HLA-A, HLA-B) or HLA class II (HLA-DR or HLA-DQ) antibodies. These were further analyzed with a Luminex Single Antigen Assay using the LABScreen HLA class I and class II antigen beads and HLA Fusion software (One Lambda, West Hills, CA, USA). The presence of DSA was determined by comparing various HLA specificities with the donor HLA type.

### 2.5. Statistical Analyses

All statistical analyses were performed using SPSS V25.0 (SPSS Inc., Chicago, IL, USA) and GraphPad Prism version 8.0.2 (GraphPad, Inc., La Jolla, CA). The one-way ANOVA or Kruskal-Wallis *H* test was used for normal distribution and abnormal distribution, respectively. If significance was found, the least significant difference or Mann–Whitney *U* test was performed to determine the difference between the groups. The chi-square test was used to evaluate basic clinical information. Correlation analyses between Tfh cell phenotypes, B cell subsets, and eGFR were conducted using Pearson or Spearman tests. The relationship between the indicators and CAMR was evaluated using a logistic regression model (forward, LR). A receiver operating characteristic (ROC) curve was used to evaluate the diagnostic performance. The optimal cut-off value was determined using the Youden index (Youden index = sensitivity + specificity − 1). All statistical tests were 2-tailed, and *p* < 0.05 was considered statistically significant.

## 3. Results

### 3.1. Demographic and Clinical Characteristics of Study Subjects

Table 1 summarizes the clinical characteristics of the 36 patients who underwent transplantation. There were no significant differences in age, BMI, sex, posttransplantation time, total HLA mismatches, or HLA antibodies among the CAMR, IgAN, and stable renal function groups. All the patients were treated with tacrolimus- (TAC-) based triple therapy (TAC + mycophenolate mofetil (MMF) + prednisone (Pred)). At the time of transplantation, some patients received induction therapy with anti-CD25mAb, basiliximab, or antithymocyte globulin. Other transplant recipients did not receive any induction therapies. Significant differences were evident in the three groups concerning renal function indicators, such as eGFR, serum creatinine, and cystatin C. The stable group had better renal function than the CAMR group (*p* < 0.05).

### 3.2. The Percentage and Absolute Number of Tfh Cell Subsets in CAMR, IgAN, and Stable Groups

**Figure 2:**
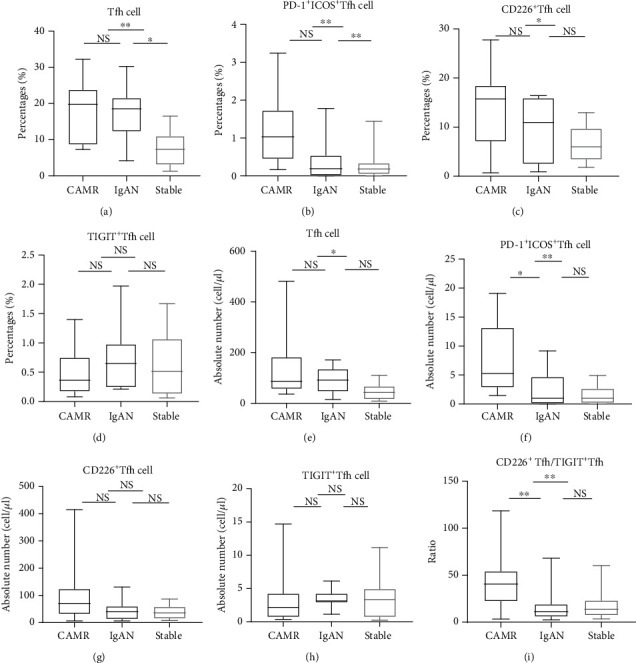
The percentage and absolute number of different Tfh phenotypes in the CAMR, IgAN, and stable groups. Percentages of (a) Tfh cells, (b) PD-1^+^ICOS^+^Tfh cells, (c) CD226^+^Tfh cells, and (d) TIGIT^+^Tfh cells. Absolute number of (e) Tfh cells, (f) PD-1^+^ICOS^+^Tfh cells, (g) CD226^+^Tfh cells, and (h) TIGIT^+^Tfh cells. (i) The ratio of CD226^+^Tfh cells to TIGIT^+^Tfh cells.

The CAMR group had the highest percentage of circulating Tfh cells (CD4^+^CXCR5^+^ T cells) (Figure 2). There were significant differences in both the percentage and number of Tfh cells between the CAMR group and stable group, and only the percentage of Tfh cells was significantly different between the IgAN and stable groups (Figures 2(a) and 2(e)). We measured the frequency and the absolute number of PD-1^+^ICOS^+^Tfh cells (PD-1^+^ICOS^+^CD4^+^CXCR5^+^ T cells), which are recognized as active Tfh cells [29]. Both the frequency and number of the PD-1^+^ICOS^+^Tfh cells were significantly higher in the CAMR group (Figures 2(b) and 2(f)). Only the frequency of CD226^+^Tfh cells (CD226^+^CD4^+^CXCR5^+^ T cells) was significantly higher in the CAMR group than in the IgAN and stable groups, whereas there were no differences between the IgAN and stable group (Figures 2(c) and 2(g)). There were no differences in the percentage and absolute number of TIGIT^+^Tfh cells (TIGIT^+^CD4^+^CXCR5^+^ T cells) among the three groups (Figures 2(d) and 2(h)). We also calculated the ratio of CD226^+^Tfh cells to TIGIT^+^Tfh cells. The ratio was significantly higher in the CAMR group than in the other two groups, while no significant difference between the IgAN and stable groups was found (Figure 2(i)).

### 3.3. The Percentage and Absolute Number of B Cell Subsets in CAMR, IgAN, and Stable Groups

**Figure 3:**
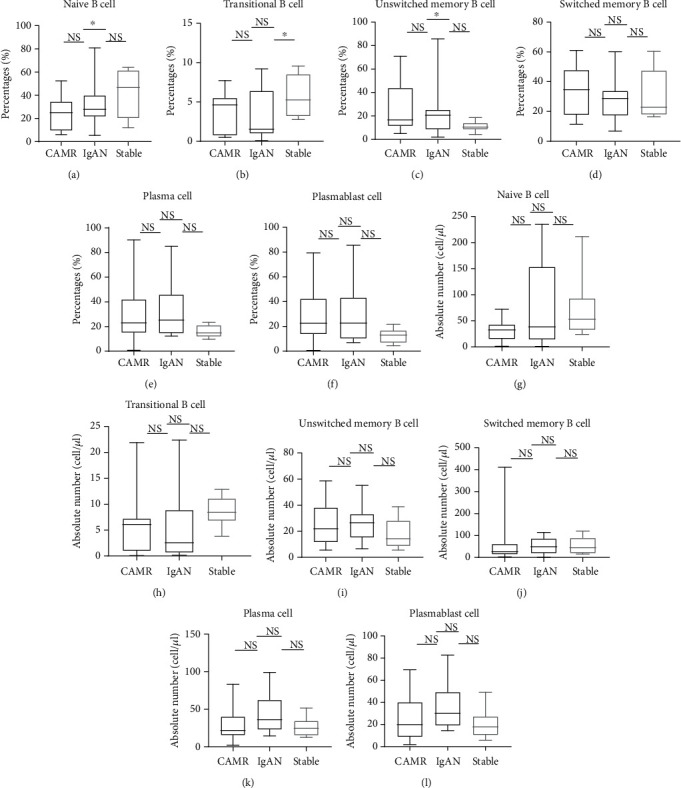
B cell differentiation subsets in patients with CAMR, recurrent de novo IgAN, and stable renal function. Percentages of (a) naïve B cells, (b) transitional B cells, (c) unswitched memory B cells, (d) switched memory B cells, (e) plasma cells, and (f) plasmablasts. Absolute numbers of (g) naïve B cells, (h) transitional B cells, (i) unswitched memory B cells, (j) switched memory B cells, (k) plasma cells, and (l) plasmablasts.

We investigated peripheral B cell subsets in different pathological types. The frequency of naïve B cells was significantly lower in the CAMR group compared to the stable group (*p* < 0.05, Figure 3(a)). The transitional B cell content differed significantly between the IgAN and stable groups (*p* < 0.05, Figure 3(b)). The percentage of unswitched memory B cells was significantly different between the CAMR and stable groups (*p* < 0.05; Figure 3(c)). No significant differences were found in either the percentage or absolute number of other B cell subsets, such as plasma cells, switched memory B cells, and plasmablasts.

### 3.4. Correlation between Tfh, B Cell Subsets, and eGFR

**Figure 4:**
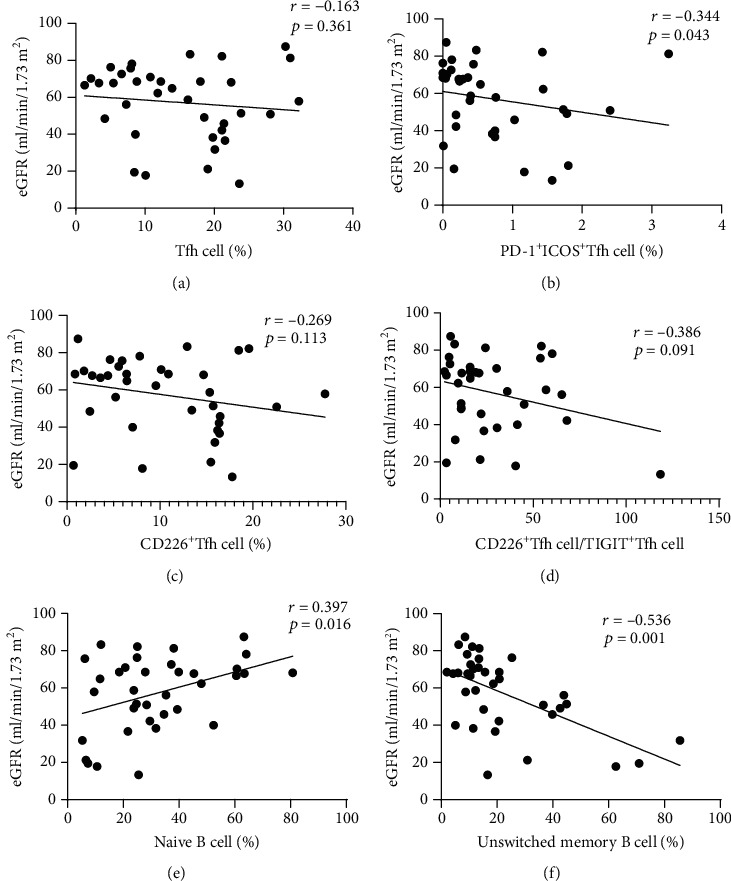
Correlation between different Tfh phenotypes, B cell subsets, and eGFR.

To explore the correlation between Tfh, B cell subsets, and renal function, we analyzed the correlation between previously statistically significant cells and eGFR. eGFR was significantly negatively correlated with PD-1^+^ICOS^+^Tfh cells (*r* = −0.344, *p* = 0.043, Figure 4(b)). eGFR was significantly positively correlated with naïve B cell levels (*r* = 0.397, *p* = 0.016, Figure 4(e)). Finally, eGFR was significantly negatively correlated with unswitched memory B cell levels (*r* = −0.536, *p* = 0.001, Figure 4(f)). Although there were no significant differences between other Tfh cell phenotypes and eGFR, a trend of a negative correlation of eGFR with other Tfh cell phenotypes was evident (Figures 4(a), 4(c), and 4(d)).

### 3.5. PD-1^+^ICOS^+^Tfh Cells, CD226^+^Tfh Cell/TIGIT^+^Tfh Cell Ratio, and eGFR Were Independent Risk Indicators for CAMR

Data on eGFR, Cys-c, the percentage and absolute number of Tfh cells and PD-1^+^ICOS^+^Tfh cells, percentage of CD226^+^Tfh cells, ratio of CD226^+^Tfh cells to TIGIT^+^Tfh cells, and percentage of naïve B cells and unswitched memory B cells were incorporated into a logistic regression model (forward: LR). The analysis identified the absolute number of PD-1^+^ICOS^+^Tfh cells, ratio of CD226^+^Tfh cells to TIGIT^+^Tfh cells, and eGFR as independent risk indicators for CAMR (odds ratio (OR) = 1.444, *p* = 0.018; OR = 1.150, *p* = 0.024; and OR = 0.880, *p* = 0.039, respectively; Table 2).

### 3.6. The ROC Curves for Diagnosing CAMR

**Figure 5:**
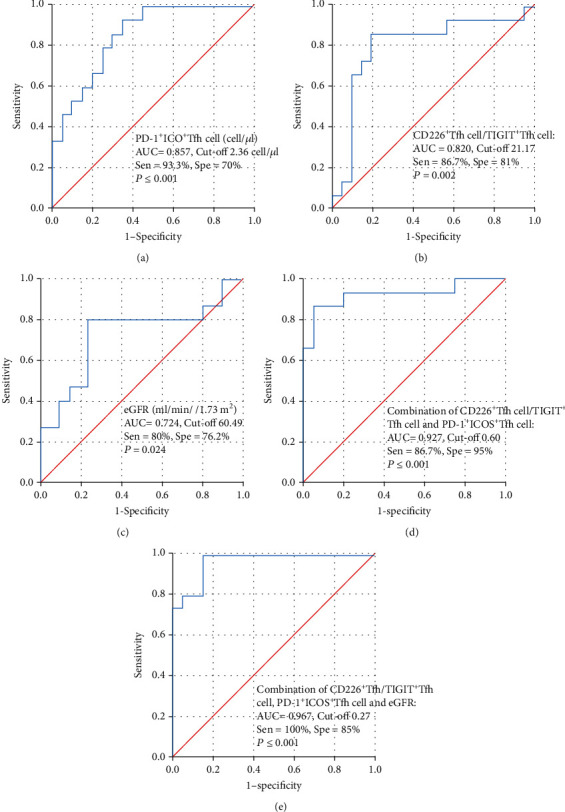
ROC curves for diagnosis of CAMR. (a) ROC curve of PD-1^+^ICOS^+^Tfh cells. (b) ROC curve of CD226^+^Tfh cell/TIGIT^+^Tfh cell ratio. (c) ROC curve of eGFR. (d) Combination of CD226^+^Tfh cell/TIGIT^+^Tfh cell ratio and PD-1^+^ICOS^+^Tfh cells. (e) ROC curve of combination of CD226^+^Tfh cell/TIGIT^+^Tfh cell ratio, PD-1^+^ICOS^+^Tfh cells, and eGFR.

ROC curves were developed to evaluate the efficacy of the absolute numbers of PD-1^+^ICOS^+^Tfh cells, CD226^+^Tfh cell/TIGIT^+^Tfh cell ratio, and eGFR. The sensitivity, specificity, and area under the ROC curve (AUC) of the absolute number of PD-1^+^ICOS^+^Tfh cells were 93.3%, 70%, and 0.857, respectively (*p* ≤ 0.001, Figure 5(a)). The sensitivity, specificity, and AUC of CD226^+^Tfh cell/TIGIT^+^Tfh cell ratio were 86.7%, 81%, and 0.820, respectively (*p* = 0.002, Figure 5(b)). The sensitivity, specificity, and AUC of eGFR were 80%, 76.2%, and 0.724, respectively (*p* = 0.024, Figure 5(c)). Based on the eGFR, chronic allograft dysfunction could be readily detected. To distinguish the different pathological types, we plotted ROC curves of PD-1^+^ICOS^+^Tfh cells and the ratio of CD226^+^Tfh cells to TIGIT^+^Tfh cells between the CAMR and IgAN groups. The results showed that PD-1^+^ICOS^+^Tfh cells and CD226^+^Tfh cell/TIGIT^+^Tfh cells both had good diagnostic efficiency for CAMR between different pathological types (Supplementary Material 1). Because a good biomarker for diagnosing CAMR in kidney transplantation should have both good sensitivity and specificity, the combination of the absolute number of PD-1^+^ICOS^+^Tfh cells and CD226^+^Tfh cell/TIGIT^+^Tfh cell ratio was considered. The regression equation was logit (*p*) = 0.387 × PD − 1^+^ICOS^+^Tfh cells (cell/*μ*l) + 0.08 × CD226^+^Tfh cell/TIGIT^+^Tfh cell − 4.058 (Supplementary Material 1). The sensitivity, specificity, and AUC of the combination of these two indicators were 86.7%, 95%, and 0.927, respectively (*p* ≤ 0.001; Figure 5(d)). We also calculated the combination of the three risk factors of sensitivity, specificity, and AUC (100%, 85%, and 0.967, respectively; *p* ≤ 0.001, Figure 5(e)). The regression equation was logit (*p*) = 0.367 × PD − 1^+^ICOS^+^Tfh cell (cell/*μ*l) + 0.140 × CD226^+^Tfh cell/TIGIT^+^Tfh cell − 0.128 × eGFR + 1.241 (Supplementary Material 1).

## 4. Discussion

We investigated the different Tfh cell phenotypes and B cell subsets in kidney transplant recipients with CAMR, de novo or recurrent IgAN, and stable renal function. Significant increases in PD-1^+^ICOS^+^Tfh cells and the ratio of CD226^+^Tfh cells to TIGIT^+^Tfh cells were evident in CAMR. We also found that stable recipients had higher levels of naïve B cells and lower unswitched memory B cells, which were also significantly related to renal function. Logistic regression analysis revealed the absolute number of PD-1^+^ICOS^+^Tfh cells, ratio of CD226^+^Tfh cells to TIGIT^+^Tfh cells, and eGFR as independent risk factors for CAMR. The combination of PD-1^+^ICOS^+^Tfh cells and the ratio of CD226^+^Tfh cells to TIGIT^+^Tfh cells showed better diagnostic efficacy for CAMR than each single parameter. The combination of all the risk factors was most sensitive for diagnosing CAMR.

Tfh cell-mediated humoral alloreactivity induces B cell differentiation and immunoglobulin production [8]. Specifically, a high level of Tfh cells was found in kidney transplant recipients with CAMR. This cell defect has been associated with a low incidence of de novo DSA [30]. A study also showed that Tfh cells could be used as biomarkers for humoral alloreactivity before the detection of alloantibodies. The authors also described that the costimulation blockade of PD-1^+^ICOS^+^Tfh cells could prevent the development of DSA [15]. Similarly, Danger et al. found that PD1^+^ICOS^+^ circulating Tfh cells were associated with de novo DSA after kidney transplantation and suggested the importance of monitoring PD1^+^ICOS^+^ circulating Tfh cells [31]. We observed that Tfh and PD-1^+^ICOS^+^Tfh cells, which are recognized as activated Tfh cell subsets [29], were both increased in CAMR. Concerning B cell subsets, kidney transplant recipients with increased levels of transitional B cells had a better clinical outcome, and transplant rejection was associated with an increase in memory-switched B cells [6, 32]. We found the same trend in B cell subsets. However, a definitive conclusion will require a study with more patients. We did detect a correlation between naïve B cells and unswitched memory B cells and renal function. The collective findings indicate that monitoring the different Tfh cell phenotypes and B cell subsets might be useful to detect immune status after kidney transplantation.

TIGIT is an inhibitory receptor that limits the adaptive immune response, whereas CD226 is involved in the positive regulation of the immune response [24]. TIGIT and CD226 expressions are increased after CD4^+^ T cell activation, and TIGIT competes with CD226 for the same ligand to inhibit cell proliferation. CD226 can suppress the proliferation of T regulatory cells (Treg) to regulate the suppression of Treg-mediated autoimmune response; CD226 deficiency can promote Treg proliferation [33]. Blocking CD226 in vitro reportedly reduced the response of allospecific T cells, although it did not reduce allospecific cytotoxicity in renal tubular epithelial cells [34]. TIGIT can be rapidly induced by antigens and other inflammatory stimuli. Therefore, TIGIT is an attractive target for immunotherapy [35]. In the field of transplantation, studies have shown that blocking the CD226-CD155 interaction by using an anti-CD226 antibody could significantly improve graft-versus-host disease (GVHD). Treatment with TIGIT-FC improved symptoms and prolongs survival, even after the onset of GVHD [36]. A phenomenon known as donor-specific hyporesponsiveness (DSH) has been described in some kidney transplant recipients and is associated with a good long-term prognosis. A study described the novel finding that TIGIT-expressing donor-reactive CD4^+^ T cells decreased several years after kidney transplantation, which could explain the development of DSH [37]. We investigated the populations of CD226^+^Tfh and TIGIT^+^Tfh cells and calculated the ratio of CD226^+^Tfh cells to TIGIT^+^Tfh cells. We observed a lower level of TIGIT^+^Tfh cells, higher level of CD226^+^Tfh cells, and increased CD226^+^Tfh cell/TIGIT^+^Tfh cell ratio in CAMR than in the stable recipients. The data support the view that the ratio of CD226^+^Tfh cells to TIGIT^+^Tfh cells is a potential biomarker and target for diagnosing or treating CAMR.

There are some limitations to this study. First, the conclusions need to be validated with larger numbers of cases and different pathological types. Second, we only compared the population in different pathological groups. No experiments explored the functions of these cells. Thus, we can only speculate on the roles of these immune parameters. Finally, although we adopted a stepwise logistic regression model, it is possible that a slight collinearity influence affected the results. Currently, biopsy is the gold diagnostic criterion. There are no standard and efficient therapies for CAMR [38]. Based on our findings, we calculated the diagnostic efficacy of PD-1^+^ICOS^+^Tfh cells, CD226^+^Tfh cell/TIGIT^+^Tfh cell ratio, eGFR, the combination of PD-1^+^ICOS^+^Tfh cells and CD226^+^Tfh cell/TIGIT^+^Tfh cell ratio, and the combination of all three indicators. The combination of PD-1^+^ICOS^+^Tfh cells and CD226^+^Tfh cell/TIGIT^+^Tfh cell ratio showed both good sensitivity and specificity and might be a potential biomarker for diagnosing CAMR. At present, there is no better way than biopsy to distinguish the specific causes of graft failure. However, the number of PD-1^+^ICOS^+^Tfh cells and CD226^+^Tfh cell/TIGIT^+^Tfh cell ratio is still effective in diagnosing CAMR only in different pathological types. In addition, the combination of all risk factors showed the best sensitivity for diagnosing CAMR, highlighting the importance of monitoring activated Tfh cells. With further studies, these checkpoints may become reliable targets to treat CAMR.

## 5. Conclusion

Our findings suggest that monitoring different Tfh phenotypes and B cell subsets is beneficial for kidney transplant recipients. Furthermore, the combination of the number of PD-1^+^ICOS^+^Tfh cells and the ratio of CD226^+^Tfh cells to TIGIT^+^Tfh cells may be a potential biomarker for diagnosing CAMR. Whether this method can be applied clinically requires further research.

## Figures and Tables

**Table 1:** Demographic and clinical characteristics of the study population.

	CAMR group (*n* = 15)	IgAN group (*n* = 11)	Stable group (*n* = 10)	*p* value
Age (years)	41.27 ± 7.00	36.36 ± 13.03	42.40 ± 6.13	0.267
BMI (kg/m^2^)	23.53 ± 4.11	22.01 ± 3.45	22.22 ± 2.66	0.533
Male	11 (73.3%)	4 (36.3%)	8 (80.0%)	0.070
Female	4 (26.7%)	7 (63.63%)	2 (20.0%)	
Posttransplantation time (years)	6 (3, 9)	6 (3, 9)	6.5 (3.75, 8.25)	0.912
PRA (%)	3.19 (0.00, 3.55)	0.40 (0.00, 0.00)	2.02 (0.00, 3.95)	0.295
Induction therapy	*n* = 13	*n* = 9	*n* = 9	0.823
Anti-CD25 mAb	3	1	1	
Basiliximab	1	3	2	
Antithymocyte globulin	4	2	2	
Total HLA mismatches	4 (4, 5)	4 (3, 4)	4 (4, 6)	0.055
Anti-HLA class I positive	2 (*n* = 12)	0 (*n* = 8)	2 (*n* = 5)	0.418
Anti-HLA class II positive	7	1	1	0.054
DSA positive	7	1	0	0.073
eGFR (ml/min/1.73 m^2^)	50.91 (21.19, 58.68)^a^	57.02 (40.79, 68.51)	69.40 (67.42, 74.03)	0.003^∗^
Scr (*μ*mol/l)	138.0 (110.0, 292.0)^a^	122 (101, 127)	106.5 (93.5, 113.0)	0.008^∗^
Cys-c (mg/l)	1.66 (1.40, 3.25)^a^	1.32 (1.21, 1.67)	1.27 (1.17, 1.35)	0.003^∗^

Data are shown as mean (SD) or median (interquartile range). Abbreviations: BMI: body mass index; Scr: serum creatinine; PRA: panel reactive antibodies, anti-CD25 mAb, and anti-CD25 monoclonal antibody; HLA: histocompatibility antigen; DSA: donor-specific antibody; eGFR: estimated glomerular filtration rate; Cys-c: cystatin C. *p* < 0.05, indicated by an asterisk (^∗^). ^a^*p* < 0.05, CAMR group vs. stable group.

**Table 2:** Logistic regression analysis for CAMR.

	Regression coefficient (*B*)	*p*	OR	95.0% CI	
				Lower	Upper
PD-1^+^ICOS^+^Tfh cell (cell/*μ*l)	0.367	0.018	1.444	1.065	1.958
CD226^+^Tfh cell/TIGIT^+^Tfh cell	0.140	0.024	1.150	1.019	1.299
eGFR (ml/min/1.73m^2^)	-0.128	0.039	0.880	0.779	0.994

## Data Availability

The datasets used and/or analyzed during the current study are available from the corresponding authors upon reasonable request.

## Supplementary Materials

Supplementary Materials ROC curve for diagnosing CAMR between the CAMR and IgAN groups, and logistic analysis of the combination of indicators.
