# Supplementary material for: The Ratio of CD226 and TIGIT Expression in Tfh and PD-1+ICOS+Tfh Cells Are Potential Biomarkers for Chronic Antibody-Mediated Rejection in Kidney Transplantation
Source: J Immunol Res. 2022 Jun 12;2022:5326083. doi: 10.1155/2022/5326083 (PMC9206998; doi:10.1155/2022/5326083)
Supplement: Supplementary Materials — ROC curve for diagnosing CAMR between the CAMR and IgAN groups, and logistic analysis of the combination of indicators. [file 5326083.f1.docx]

ROC curve for diagnosing CAMR between CAMR group and IgA group.

|  | AUC | Cut-off value | sensitivity | specificity | p-value |
| --- | --- | --- | --- | --- | --- |
| PD-1^+^ICOS^+^ Tfh cell (cell/μl) | 0.820 | 2.36 | 86.7% | 70% | 0.008 |
| CD226^+^ Tfh cell/TIGIT^+^ Tfh cell | 0.818 | 20.28 | 86.7% | 81.8% | 0.006 |

Logistic analysis of combination of PD-1+ICOS+ Tfh cell，CD226+ Tfh cell/TIGIT+ Tfh cell

|  | **Regression coefficient（B）** | | **P** | **OR** | **95.0% CI** | |
| --- | --- | --- | --- | --- | --- | --- |
|  |  |  |  |  | **Lower** | **Upper** |
| **PD-1+ICOS+ Tfh cell (cell/μl)** | 0.387 | 0.028 | | 1.472 | 1.043 | 2.077 |
| **CD226+ Tfh cell/TIGIT+ Tfh cell** | 0.080 | 0.010 | | 1.084 | 1.019 | 1.152 |
| **constant** | -4.058 | 0.002 | | 0.017 |  |  |

Logistic analysis of combination of PD-1^+^ICOS^+^ Tfh cell，CD226^+^ Tfh cell/TIGIT^+^ Tfh cell and eGFR (ml/min/ /1.73m^2^)

|  | **Regression coefficient（B）** | | **P** | **OR** | **95.0% CI** | |
| --- | --- | --- | --- | --- | --- | --- |
|  |  |  |  |  | **Lower** | **Upper** |
| **PD-1^+^ICOS^+^ Tfh cell (cell/μl)** | 0.367 | 0.018 | | 1.444 | 1..065 | 1.958 |
| **CD226^+^ Tfh cell/TIGIT^+^ Tfh cell** | 0.140 | 0.024 | | 1.150 | 1.019 | 1.299 |
| **eGFR (ml/min/ /1.73m^2^)** | -0.128 | 0.062 | | 0.880 | 0.779 | 0.994 |
| **constant** | 1.241 | 0.546 | | 3.460 |  |  |
